# Early and Appropriate Use of Ceftazidime-Avibactam in the Management of Multidrug-Resistant Gram-Negative Bacterial Infections in the Indian Scenario

**DOI:** 10.7759/cureus.28283

**Published:** 2022-08-22

**Authors:** Subramanian Swaminathan, Abhisek Routray, Akshata Mane

**Affiliations:** 1 Infectious Disease, Gleneagles Global Hospitals, Chennai, IND; 2 Medical Affairs, Pfizer Limited, Mumbai, IND

**Keywords:** rapid diagnosis, gram-negative bacteria, carbapenem resistance, early use, ceftazidime-avibactam

## Abstract

The increasing prevalence of antibiotic-resistant pathogens exerts a substantial burden on the healthcare infrastructure worldwide. The World Health Organization (WHO) has declared that multidrug-resistant (MDR) Gram-negative pathogens, especially, carbapenem-resistant *Enterobacterales* (CRE), *Acinetobacter baumannii*, and *Pseudomonas aeruginosa* as the topmost priority while developing newer antimicrobials. The increasing prevalence of infectious diseases caused by MDR Gram-negative bacteria also poses a challenge when choosing the empiric antimicrobial therapy for seriously ill hospitalized patients. The infections caused by MDR Gram-negative organisms ultimately result in increased mortality, morbidity, prolonged hospital stay, and increased cost of management. To tackle these challenges, newer antimicrobials like ceftazidime-avibactam were explored. The article also discusses the *in vitro* activity and therapeutic efficacy of ceftazidime-avibactam along with its pharmacokinetic properties and the role it will play in the management of MDR Gram-negative organisms in the Indian setting. Several studies have highlighted the role of early and appropriate antibiotic use in the reduction of mortality in patients with Gram-negative infections. Timely initiation of appropriate antibiotic therapy for serious infections leads to favorable clinical outcomes. Early and appropriate use of ceftazidime-avibactam while treating MDR Gram-negative infections has been associated with improved clinical outcomes. The aim of this review is to highlight the efficacy of ceftazidime-avibactam in the treatment of MDR Gram-negative infections. We have also summarized the information on outcomes achieved by early and appropriate use of ceftazidime-avibactam.

## Introduction and background

The emerging widespread antibiotic-resistant pathogens exert a significant burden on the healthcare infrastructure. The multidrug-resistant (MDR) Gram-negative pathogens including carbapenem-resistant *Enterobacterales* (CRE), *Acinetobacter baumannii*, and *Pseudomonas aeruginosa* are considered by the World Health Organization (WHO) as the highest priority while developing newer antimicrobials [1,2]. The carbapenem resistance in *Enterobacterales* is driven by carbapenemases such as New Delhi metallo-β-lactamase (NDM), and Oxacillinase-48 like (OXA-48-like) VIM, IMP and KPC [3,4]. In India, high carbapenem resistance among *Enterobacterales* has been reported by the Indian Council of Medical Research (ICMR) with resistance rates up to 30% for *Escherichia coli *and 50% for *Klebsiella pneumoniae* [5]. OXA-48-like gene was identified in 52% of the carbapenem-resistant (CR)-*K. pneumoniae* isolates while 20% isolates possessed NDM gene and 27% isolates had both NDM with OXA-48-like gene. However, in carbapenem-resistant (CR)-*E. coli*, NDM was identified in 68% of isolates followed by OXA-48-like in 24% isolates and 8% isolates carried both NDM with OXA-48-like gene [5].

The increasing prevalence of infectious diseases caused by MDR Gram-negative bacteria places a hurdle in the selection of appropriate empiric antimicrobial therapy for seriously ill hospitalized patients [2]. The management of infections caused by CREs is more challenging owing to limited antimicrobial options. CREs exhibit resistance against conventional first-line antimicrobials including cephalosporins, β-lactam-β-lactamase inhibitors, carbapenems, and fluoroquinolones [6].

Infections caused by MDR organisms are responsible for increased mortality, morbidity, prolonged hospital stay, and increased cost of management [7]. They pose a serious threat to the healthcare infrastructure owing to their difficult management. Early detection of MDR organisms can facilitate the start of appropriate antibiotic treatment and better therapeutic decisions to ensure favorable clinical outcomes and survival rates [7]. Early diagnosis can thus help in implementing improved patient management strategies and appropriate antibiotic use.

There is no concrete consensus for optimal regimens in various guidelines or experts’ opinions. Colistin and tigecycline have been used as first-line therapy for managing infections caused by CREs [8]. However, tigecycline does not attain the required plasma concentrations, and hence may not be used for treating bloodstream infections [5]. Colistin has been associated with prominent toxicity (both nephrotoxicity and neurotoxicity), which may limit its clinical use [8]. Hence, these two regimens can be avoided since newer treatment modalities are available. These challenges have led to the development of newer antimicrobials such as ceftolozane-tazobactam, imipenem-cilastatin-relebactam, plazomicin, meropenem-vaborbactam, ceftazidime-avibactam, eravacycline, and cefiderocol [8].

Ceftazidime-avibactam, a combination of the third-generation cephalosporin ceftazidime and non-β-lactam-β-lactamase inhibitor avibactam, is an intravenously administered antimicrobial [9]. Ceftazidime-avibactam is indicated for the treatment of following infections in adults i.e. complicated intra-abdominal infection (cIAI), complicated urinary tract infection (cUTI), including pyelonephritis, hospital-acquired pneumonia (HAP) including ventilator-associated pneumonia (VAP) with susceptible Gram-negative microorganism and treatment of patients with bacteremia that occurs in association with, or is suspected to be associated with, any of the infections mentioned above [10]. Ceftazidime-avibactam has been proven to be clinically efficacious in pivotal phase III non-inferiority trials in comparison with carbapenems [11-13]. The *in vitro* activity of ceftazidime-avibactam has been established against extended-spectrum β-lactamase (ESBLs), AmpC β-lactamase, *Klebsiella pneumoniae* carbapenemase (KPC), and OXA-48 producing *Enterobacterales *and *Pseudomonas aeruginosa* isolates [9]. Real-world evidence globally has demonstrated the effectiveness and safety of ceftazidime-avibactam in the management of MDR Gram-negative infections including CRE. Similarly, a few real-world evidence studies have published data supporting the use of ceftazidime-avibactam to treat MDR Gram-negative infections in Indian patients.

The aim of this review is to highlight the efficacy of ceftazidime-avibactam in the management of MDR Gram-negative infections. The article also discusses the* in vitro* activity and therapeutic efficacy of ceftazidime-avibactam along with its pharmacokinetic properties and the role it will play in the management of MDR Gram-negative organisms in the Indian setting.

## Review

Methodology

An in-depth literature search was conducted using PubMed and Google Scholar databases. There were no predefined inclusion/exclusion criteria for article selection. The articles were selected on the basis of their suitability and relevance to the pre-decided outline by the authors.

Mechanism of action

Ceftazidime prevents bacterial cell wall synthesis which causes bacterial cell death [14]. It binds to penicillin-binding proteins (PBPs) of Gram-negative bacteria which decreases the cross-linking activity of peptidoglycan and leads to the inhibition of cell wall synthesis [15]. Avibactam is a non-β-lactam, β-lactamase inhibitor which causes covalent acylation of the β-lactamase to inactivate susceptible β-lactamases [9]. This structure is not hydrolyzed and is slowly separated. Avibactam is then reversed to its original structure. Avibactam does not possess antibacterial properties [16]. *In vitro*, avibactam exhibits activity against Ambler (a classification system for β-lactamases) class A, including TEM, SHV, CTX-M, KPC, GES, PER, SME; plasmid class C including FOX, MOX, CMY, LAT, ACC, DHA and chromosomal class C (AmpC); and class D including OXA-48 [16]. Additionally, inhibitors such as sulbactam and tazobactam are penicillin-based sulfones and lack the ability to inhibit carbapenemases [17]. Avibactam does not inhibit class B metallo-β-lactamases (NDM, VIM, IMP, VEB, PER), and OXA-23 and OXA-24/40 carbapenemases [17].

*In vitro* activity

*In vitro *activity of ceftazidime-avibactam was analyzed on isolates collected from nine centers across India between 2018 to 2019, as a part of the ATLAS (Antimicrobial Testing Leadership and Surveillance) program. The *in vitro* activity of ceftazidime-avibactam and comparator drugs was analyzed against *E. coli* (n = 458) and *K. pneumoniae *(n = 455) isolates. An overall susceptibility rate of over 70% was reported among *K. pneumoniae* and *E. coli* isolates. Around 51% of carbapenem-resistant (CR)-*K. pneumoniae* and 24% of CR-*E. coli* isolates were found to be susceptible to ceftazidime-avibactam [5]. 

The global ATLAS data collected between 2012 and 2016 reported *in vitro *susceptibilities of Gram-negative isolates against ceftazidime-avibactam. The study reported susceptibility of CR-*E. coli* to be 72.3% using Clinical Laboratory and Standards Institute (CLSI) breakpoints against ceftazidime-avibactam. Similarly, susceptibility of CR-*K. pneumoniae* was reported to be 85.6% using CLSI breakpoints [18].

Pharmacokinetics

The steady-state volumes of distribution of ceftazidime and avibactam were approximately 22 and 18 L. The human protein binding of both ceftazidime and avibactam is approximately 10% and 8%, respectively [9]. Around 80-90% of the injected ceftazidime is eliminated by the kidneys in its unchanged form with the renal clearance of 115 mL/min. Avibactam is excreted via the urine without alteration and has a renal clearance of ~158 mL/min. In healthy adults, with normal renal function, the half-life (t1/2) of both ceftazidime and avibactam is 2.76 hours and 2.71 hours, respectively [19]. Dose adjustment is required in patients with mild, moderate, or severe renal impairment to avoid an accumulation of the drug [20]. Neither ceftazidime nor avibactam is observed to undergo significant hepatic metabolism. The potential of drug-drug interactions is low for ceftazidime-avibactam. Patients with hepatic impairment require no dose adjustments. Age, weight, gender, or ethnicity do not impact the pharmacokinetics of ceftazidime-avibactam therefore dosage adjustment is not essential [9].

Compartmental pharmacokinetic studies have shown that ceftazidime (52%), as well as, avibactam (42%) penetrate into epithelial lining fluid (ELF), which is greater than previously calculated at plasma concentrations relevant for efficacy (~ 8 mg/l for ceftazidime and ~ 1 mg/L for avibactam). These results suggest epithelial lining fluid (ELF) exposures of both drugs exceeded levels required for efficacy in plasma [21,22]. The efficacy of an antibiotic to successfully mitigate pneumonia depends on the concentration of the unbound drug available at the pulmonary infection site [22]. 

Therapeutic efficacy

Landmark Trials

The efficacy and safety profile of ceftazidime-avibactam has been studied by a number of randomized, multicenter, controlled phase III trials. In all studies, patients assigned to ceftazidime-avibactam received a combination of 2.5 g ceftazidime-avibactam as a two-hour intravenous infusion every eight hours. Dose adjustment was applied for patients with moderate renal impairment at baseline (estimated Cockcroft-Gault-calculated creatinine clearance {CrCl}, >30 to ≤50 mL/min). Most of these trials had separate primary endpoints based on US Food and Drug Administration (FDA) guidance; and European Medicines Agency (EMA) guidance (Table 1) [9].

**Table 1:** Efficacy and Safety Profile of Ceftazidime- Avibactam and Comparators from the Phase III Clinical Trials CAZ-AVI: Ceftazidime-avibactam; cIAI: complicated intra-abdominal infections; cUTI: complicated urinary tract infections; HAP: hospital-acquired pneumonia; VAP: ventilator-associated pneumonia; mMITT: microbiological modified intent-to-treat; cMITT: clinically modified intent-to-treat; CE: clinically evaluable; CI: confidence interval RECAPTURE: Ceftazidime-avibactam Versus Doripenem for the Treatment of Complicated Urinary Tract Infections, Including Acute Pyelonephritis; RECLAIM: Efficacy and Safety of Ceftazidime-Avibactam Plus Metronidazole Versus Meropenem in the Treatment of Complicated Intra-abdominal Infection; REPRISE: Replicating Evidence of Preserved Renal Function: an Investigation of Tolvaptan Safety and Efficacy in ADPKD; REPROVE: Ceftazidime-avibactam versus meropenem in nosocomial pneumonia, including ventilator-associated pneumonia BAT: Best available therapy was determined by the investigator on the basis of standard of care and local label recommendations and was documented before randomization. Preferred best available therapy options for complicated urinary tract infection and complicated intra-abdominal infection were 5–21 days of treatment with meropenem, imipenem, doripenem, colistin, and (for complicated intra-abdominal infection) tigecycline, administered intravenously, but any therapy, including combination treatment, was permitted. REPRISE [23]; RECAPTURE - 1 and -2 [24]; RECLAIM - 1 and -2 [25]; RECLAIM Indian subset [26]; RECLAIM -3 [27]; REPROVE [13]; REPROVE Indian subset [28]; Pooled data of RECAPTURE-1 and -2, RECLAIM-1 and -2 and REPRISE trials [9];

Sr.No	Trial name	Indication	Treatment	Primary Outcome	Conclusion
1	REPRISE	cIAI/ cUTI, including pyelonephritis	cIAI: CAZ-AVI + Metronidazole (n=10), BAT (n=11) cUTI: CAZ-AVI (n= 144), BAT (n = 137)	Clinical cure at the test-of-cure visit (7–10 days after last infusion of study therapy) in the mMITT population*: CAZ-AVI: 91% (95% CI 85.6 to 94.7), BAT: 91% (95% CI 85.9 to 95.0) *Patients in the mMITT population are defined as carrying a pathogen at the start of treatment and who received at least one dose of study drug.	Numerically higher proportion of patients on ceftazidime – avibactam achieved a favorable microbiological response
2	RECAPTURE - 1 and -2	cUTI including pyelonephritis	CAZ-AVI (n = 516) Doripenem (n= 517)	Clinical cure at the test-of-cure visit (21–25 days after randomization) mMITT* CAZ-AVI (n = 393): 90.3% Doripenem (n = 417): 90.4% Difference, % (95% CI): -0.1 (-4.23 to 4.03) *The mMITT population comprised all randomized patients with minimum disease criteria and eligible baseline pathogen(s).	Ceftazidime-avibactam was non-inferior to doripenem in the treatment of hospitalized patients with cUTI or acute pyelonephritis
3	RECLAIM - 1 and -2	cIAI	CAZ-AVI + Metronidazole (n = 532) Meropenem (n= 534)	Clinical cure at the test-of-cure visit (28-35 days after randomization) MITT* CAZ-AVI + Metronidazole (n = 520): 82.5% Meropenem (n= 523): 84.9% Difference, % (95% CI): -2.4 (-6.90 to 2.10) *Patients in the MITT population are defined as patients who received study drug and met the clinical disease criteria.	Ceftazidime-avibactam plus metronidazole was non-inferior to meropenem
4	RECLAIM Indian subset	cIAI	CAZ-AVI + Metronidazole (n = 61 Meropenem (n = 63)	Clinical cure at TOC In mMITT population: CAZ-AVI + Metronidazole – 83.3%, meropenem – 77.1% Difference 6.2 (-14.31 to 25.65) In CE population: CAZ-AVI + Metronidazole – 97.8%, meropenem – 95.5% Difference 2.4 (-7.41 to 13.33)	Ceftazidime-avibactam can be considered as an alternative to carbapenems for treating resistant pathogens in the ICU setting The results of the study were in-line with the results of the overall results
5	RECLAIM -3	cIAI	CAZ-AVI + Metronidazole (n = 215) Meropenem (n= 217)	Clinical cure at the test-of-cure visit (28-35 days after randomization) CE CAZ-AVI + Metronidazole (n = 166): 93.8% Meropenem (n= 173): 94.0% Difference, % (95% CI): -0.2 (-5.53 to 4.97)	Ceftazidime-avibactam plus metronidazole was non-inferior to meropenem
6	REPROVE	HAP/ VAP	CAZ-AVI (n = 405) Meropenem (n = 403) cMITT population CAZ-AVI (n = 356) Meropenem (n= 370) CE population CAZ-AVI (n = 257) Meropenem (n= 270)	Clinical cure at the test-of-cure visit (21–25 days after randomization) cMITT* CAZ-AVI (n = 245): 68.8% Meropenem (n= 270): 73.0% Difference, % (95% CI): -4.2 (-10.76 to 2.46) CE** CAZ-AVI (n = 199): 77.4% Meropenem (n= 211): 78.1% Difference, % (95% CI): -0.7 (-7.86 to 6.39) *cMITT population comprised patients with minimum disease criteria but excluded patients with only non-target pathogens. **The clinically evaluable population comprised patients in the cMITT population who received an adequate course of treatment and had an assessable clinical outcome within the assessment window, no protocol deviation that could affect the assessment of efficacy, and no unacceptable previous or concomitant antibiotics.	Ceftazidime-avibactam plus metronidazole was non-inferior to meropenem
7	REPROVE Indian subset	HAP	CAZ-AVI (n = 36 Meropenem (n = 42)	Clinical cure at TOC In cMITT population: CAZ-AVI – 80.6%, meropenem – 71.8% Difference 8.9 (-12.09 to 28.27) In CE population: CAZ-AVI – 88.0%, meropenem – 77.1% Difference 10.9 (-10.39 to 29.77)	The clinical efficacy of ceftazidime-avibactam was comparable to meropenem for the management of nosocomial pneumonia The results of the study were in-line with the results of the overall results
8	Pooled data of RECAPTURE-1 and -2, RECLAIM-1 and -2 and REPRISE trials	cIAI, cUTI, VAP Nosocomial pneumonia	Enterobacteriaceae – 1051/2240 i.e., 46.9% were MDR P. aeruginosa – 95/272 i.e., 34.9% were MDR	Microbiological response at TOC for all Enterobacteriaceae: CAZ-AVI – 78.4% Comparators - 79.6% Clinical cure at TOC: CAZ-AVI - 85.4% Comparators – 86.1%	Ceftazidime-avibactam demonstrated similar clinical efficacy to predominantly carbapenem comparators against MDR pathogens

Real-World Evidence

The efficacy of ceftazidime-avibactam is corroborated by mounting clinical evidence. Several studies have been published in various types of primary infections including, but not limited to IAIs, UTIs, HAP, and VAP, and bacteraemia secondary to these [11,29-33]. A summary of the findings has been presented in Table 2. The real-world evidence is indicative of favorable efficacy results which were consistent even in MDR infections.

**Table 2:** Efficacy of Ceftazidime-Avibactam – Real World Evidence on cIAI, cUTI, pyelonephritis, HAP, VAP, and secondary bacteremia CAZ-AVI: Ceftazidime-avibactam; cIAI: complicated intra-abdominal infections; CNS: Central nervous system; CRE: Carbapenem-resistant *Enterobacterales*; cUTI: complicated urinary tract infections; HAP: Hospital-acquired pneumonia; MDR: Multi-drug resistant; MIC: Minimum inhibitory concentration; PDR: Pan-drug resistant; SSTI: Skin and soft tissue infection; VAP: Ventilator-associated pneumonia; XDR: Extremely drug-resistant Shields et al., 2016 [34]; Shields et al., 2017 [35]; Temkin et al., 2017 [36]; King et al., 2017 [37]; Van Duin et al., 2018 [38]; Santevecchi et al., 2018 [33]; Sousa et al., 2018 [39]; Alraddadi et al., 2019 [40]; Jorgensen et al., 2019 [11]; Calle et al., 2019 [41]; Tumbarello et al., 2019 [42]; Vena et al., 2020 [31]; Rathish et al., 2021 [29]; Nagvekar et al., 2021 [30]

Sr. No.	Reference	Type of Infection/s	Treatment group/s	Causative Organisms	Key Outcomes
1	Shields et al., 2016	Pneumonia - 32% (50% VAP, 50% HAP) IAI - 11% Pyelonephritis - 11%	CAZ-AVI (n = 37) Monotherapy – 26 patients	CR-*K. pneumoniae* – 84% CE-*E. coli *– 8% CR-*Enterobacter cloacae* – 5% CR-*Enterobacter aerogenes* – 3%	30-day survival rate – 76% 90-day survival rate – 62% Clinical success rate – 59% Microbiological failure – 27%
2	Shields et al., 2017	Bacteremia with CR-Kp Secondary bacteremia – 81/109	CAZ-AVI (n = 13) Carbapenem + aminoglycoside (n = 25) Carbapenem + colistin (n = 30) Others (n = 41)	CR-*K. pneumoniae*	Clinical success CAZ-AVI – 85% Carbapenem + Aminoglycoside – 48% Carbapenem + Colistin – 40% Others – 37% 30-day survival rate CAZ-AVI – 92% Carbapenem + Aminoglycoside – 68% Carbapenem + Colistin – 70% Others – 68% 90-day survival rate CAZ-AVI – 92% Carbapenem + Aminoglycoside – 56% Carbapenem + Colistin – 63% Others – 49%
3	Temkin et al., 2017	Bacteremia - 68% IAI - 39% Pneumonia - 18% UTI - 8%	CAZ-AVI (n=38) Monotherapy – 13 patients	*K. pneumoniae* (n = 34) *P. aeruginosa* (n =2) *K. oxytoca* (n = 1) *E. coli *(n = 1)	For KPC-producing Enterobacterales: Microbiological cure - 75% Clinical cure - 68% Survival to hospital discharge – 77% For OXA-48-producing Enterobacterales: Microbiological cure - 25% Clinical cure - 32% Survival to hospital discharge - 23% Clinical cure, by infection site IAI – 66.7% Pneumonia – 42.9% UTI – 66.7% Microbiological cure, by infection site IAI – 40% Pneumonia – 42.9% UTI – 66.7% Adverse events reported in 16% patients
4	King et al., 2017	UTI - 28% Pneumonia - 27% IAI - 7%	CAZ-AVI (n=60) Monotherapy: 55%	CRE infections caused by: *K. pneumonia *– 83% *E. col*i – 8% Others – 9%	In hospital mortality: 32% Microbiological cure: 53% Clinical success: 65%
5	Van Duin et al., 2018	Pneumonia - 22% UTI - 14%	CAZ-AVI ( n = 38); monotherapy – 37% Colistin (n = 99)	KPC-producing Enterobacterales	30-day adjusted all-cause-hospital mortality – CAZ-AVI: 9% Colistin: 32%
6	Santevecchi et al., 2018	Pneumonia - 46% Intra–abdominal - 15%	CAZ-AVI (n = 10) Monotherapy – 5 patients	*P. aeruginosa, Citrobacter freundii, Enterobacter aerogenes* 13/21 isolates met the Magiorakos criteria of MDR infections	Ceftazidime-avibactam median MIC was 1.5 mg/L (range 0.5–8 mg/L) Microbiological cure - 67% (n = 6/9) Clinical success - 70% (n = 7/10) All-cause mortality - 30% (n = 3/10) No adverse events reported
7	Sousa et al., 2018	Intra-abdominal - 28% Respiratory - 26% Urinary - 25%	CAZ-AVI (n = 57) Monotherapy – 46 (81%) patients	OXA-48-producing *Enterobacteriaceae*	Mortality at 14 days – 14% Recurrence rate at 90 days – 10%
8	Alraddadi et al., 2019	HAP – 36.8% cUTI – 28.9% cIAI – 21.1%	CAZ-AVI (n = 10) Comparative group (n = 28)	Carbapenem-resistant *Enterobacteriaceae K. pneumonia *– 78.9% *E. coli* – 21.1%	All-cause mortality at 30 days – 50% with CAZ-AVI and 57% with comparator
9	Jorgensen et al., 2019	Respiratory tract infections - 37% UTI - 20% IAI - 19.7%	CAZ-AVI (n = 203) Monotherapy – 68 patients	MDR Gram-negative bacteria 117 - CRE (63.2% CR*-K. pneumoniae* and 14.5% CR-*E. coli)* 63 - *Pseudomonas spp.*	Composite clinical failure and 30-day mortality: 59 (29.1%) CRE patients and 35 (17.2%) Pseudomonas spp. patients Outcomes when the treatment was initiated within 48 hours: Clinical success rate - 33.3% Clinical failure - 18.6%
10	Calle et al., 2019	Intraabdominal - 29% Urinary - 25% Respiratory - 21%	CAZ-AVI (n = 24)	OXA-48 CPE *Klebsiella pneumoniae* - 95.8% episodes *Escherichia coli* in one episode	30-day mortality – 8.3% 90-day mortality - 20.8% Clinical cure at 30 days - 62.5% of episodes
11	Tumbarello et al., 2019	Intra-abdominal – 8.7% LRTI – 9.4% Urinary – 4.3% Bloodstream – 75.4%	CAZ-AVI (n = 138)	All isolates were KPC-producing *K. pneumoniae*	Overall, 30-day mortality – 34.1% 30-day mortality in patients who received CAZ-AVI – 36.5% versus patients who were on control – 55.8%
12	Vena et al., 2020	Nosocomial pneumonia – 49% IAI – 10%	CAZ-AVI (n = 41)	45 isolates (24% MDR, 56% XDR, 20% PDR) *P. aeruginosa *(80.5%)* Enterobacterales* (9.8%)	Clinical success – 90.5% (overall) P. aeruginosa – 87.8% ESBL* Enterobacterales* – 100% Mixed infection – 100% Development of resistance – not detected No adverse events reported
13	Rathish et al., 2021	Pneumonia – 16% IAI – 10% UTI – 9%	CAZ-AVI (n = 103) Monotherapy – 69 patients	*K. pneumoniae *(48%) E. coli (4%) *P. aeruginosa* (4%) 12 patients had CRE infection	All-cause mortality – 27% Clinical cure – 73%
14	Nagvekar et al., 2021	IAI – 32% Nosocomial pneumonia – 26% Bloodstream infections – 9% cUTI – 9%	CAZ-AVI (n = 121) Monotherapy – 4 patients	119 culture-confirmed CRE isolates *K. pneumoniae* – 84.21% *E. coli *– 15.78% Resistance mechanism- OXA 48 – 33.61% NDM + OXA 48 – 37.81% NDM – 28.57%	Clinical cure rates - OXA 48 Overall – 82.35% Ceftazidime-avibactam (alone) – 100% Ceftazidime-avibactam + Polymyxin – 75% Ceftazidime-avibactam + Tigecycline –71% Ceftazidime-avibactam + Polymyxin + Fosfomycin – 100% NDM + OXA 48 OR NDM Overall – 77.5%

Early and appropriate use of ceftazidime-avibactam

Early detection of MDR organisms helps in deciding the appropriate antibiotic for the treatment of infections. Thus, facilitating better therapeutic decisions to ensure favorable clinical outcomes and survival rates. Conventional specimen culture is one such diagnosis method that is used to identify causative organisms in about 48 hours [7]. The causative organisms after isolation can be subjected to molecular testing (also known as genotyping) or phenotypic testing of bacterial antimicrobial susceptibility at the discretion of the treating physician.

Phenotypic testing includes diffusion method, dilution method, E-test, broth macro and micro dilution, and Matrix-Assisted Laser Desorption Ionization-Time of Flight Mass Spectroscopy (MALDI-ToF) [43]. Molecular testing includes polymerase chain reaction (PCR), DNA microarray and DNA chips, and loop-mediated isothermal amplification (LAMP). The newer molecular diagnostic methods generate results within one to four hours. This enables physicians to optimize a targeted treatment for the patients in a timely manner. Overall, this may lead to a decrease in mortality, shorter hospital stays, and a reduction in hospitalization costs [44-47]. Rapid diagnostics is associated with early intervention with effective antimicrobial therapy which may lead to improved clinical outcomes and decreased mortality [48].

Multiplex real-time PCR technique assay can be utilized for rapid detection of carbapenemase genes in the infection-causing isolates. It can detect five carbapenemase genes (*KPC*, *NDM*, *VIM*, *IMP-1*, and *OXA-48*) and has good concordance rates of between 90 to 100% [49]. The evolution of rapid diagnostics has reduced the time required to get the results. This has led to positive effects on clinical outcomes in patients and also has contributed to the efforts to counter antimicrobial resistance in conjunction with robust antimicrobial stewardship programs [50]. A rapid turn-around time to obtain test results leads to a shorter time to initiate optimal therapy. The subsequent advantages of using rapid diagnostic tools and antimicrobial stewardship programs include a decrease in mortality rates, shorter hospital lengths of stay, and reduced hospital costs [50]. Early diagnosis of MDR organisms can thus help in implementing improved management strategies and standardizing antimicrobial stewardship policies. The information on the turn-around time of various diagnostic tests is presented in Table 3 [44-47].

**Table 3:** Test methods detecting carbapenemase activity/specific carbapenemase gene *Detection of carbapenemase activity ^#^Detection of specific carbapenemase gene Carba NP: Carbapenemase detection; MALDI-TOF MS: Matrix-Assisted Laser Desorption Ionization-Time of Flight Mass Spectrometry; PCR: Polymerase chain reaction

Test method	Accuracy	Turn-around time
Modified Hodge test*	Moderate	Next day
Carba NP test*	Moderate	Same day
Carbapenemase inactivation method*	High	Next day
MALDI-TOF MS*	High	Same day
PCR^#^	High	Same day
Microarray^#^	High	Same day

Inappropriate use of broad-spectrum antimicrobials is responsible for increased antimicrobial resistance (AMR). It is also responsible for an increase in the rate of adverse events in up to 20% of patients [51]. The American College of Physicians (ACP) and the Centers for Disease Control and Prevention (CDC) have reported an estimate of over 2.6 million diseases and 35900 deaths annually due to AMR. They also have reported the incidence of resistant infections to be 6.1/10000 person-days after receiving antibiotics [51]. A multivariable survival analysis conducted on 789 patients suffering from *E. coli*, *Klebsiella spp.* and *P. aeruginosa *caused bacteremia, demonstrated that the patients who received an effective antibiotic early (hazard ratio {HR} - 1.26, confidence interval i.e., CI - 0.78 to 2.06) had better survival rate as compared to those who did not (HR - 1.83, CI - 1.05 to 3.20) [52]. In patients suffering from infections caused by resistant Gram-negative organisms, delayed appropriate therapy rates remain high. As a consequence, the negative impact of increased duration of therapy (+4.5 days) and delayed recovery (+4.9 days) have been observed [53]. A retrospective analysis concluded that delayed appropriate therapy is an independent factor related to unfavorable clinical outcomes as compared to timely appropriate therapy among hospitalized patients with serious infections due to Gram-negative bacteria, regardless of resistance status. The patients who received delayed appropriate therapy experienced an approximate 70% increase in length of stay, about 65% increase in total in-hospital costs and approximately 20% increase in the risk of in-hospital mortality or discharge to hospice [53]. Early and appropriate antibiotic use results in the reduction in mortality in patients with sepsis [54,55].

Several clinical studies have highlighted the importance of timely initiating antibiotic therapy for serious infections to obtain favorable clinical outcomes. The summary has been presented in Table 4 [56-58].

**Table 4:** List of studies with < 72 hours of time to initiation of ceftazidime avibactam BAT: Best available therapy; CAZ-AVI: Ceftazidime-avibactam; cIAI: complicated intra-abdominal infections; CRE: carbapenem-resistant Enterobacterales; cUTI: complicated urinary tract infections; ESBL: extended-spectrum beta-lactamase; HAP: hospital-acquired pneumonia; KPC: Klebsiella pneumoniae carbapenemase; MDR: multi-drug resistant; MIC: minimum inhibitory concentration; PDR: pan-drug resistant; SSTI: skin and soft tissue infection; VAP: ventilator-associated pneumonia; XDR: extremely drug-resistant Jorgensen et al., 2019 [11]; Calle et al., 2019 [41]; Caston et al., 2022 [59]

Sr. No.	Reference	Type of Infection/s	Treatment group/s	Time of treatment initiation	Causative Organisms	Key Outcomes
1	Jorgensen et al., 2019	Respiratory tract infections - 37% UTI - 20% IAI - 19.7%	CAZ-AVI (n = 203) Monotherapy – 68 patients	72 hours within the onset of infection	MDR Gram-negative bacteria 117 - CRE (63.2% CR-K. pneumoniae and 14.5% CR-E. coli) 63 - *Pseudomonas spp.*	Composite clinical failure and 30-day mortality: 59 (29.1%) CRE patients and 35 (17.2%) Pseudomonas spp. patients. Patients who received CAZ-AVI within 48 hours of culture collection – adjusted odds ratio – 0.409 (CI: 0.180 to 0.930)
2	Calle et al., 2019	IAI – 29% UTI – 25% Pneumonia – 21% Others – 25%	CAZ-AVI (n = 23; 24 episodes) Monotherapy – 59% episodes	First 48 hours (Mean 2.5 days)	All isolates were OXA-48 and co-produced ESBLs (except 1) *K. pneumoniae* – 95.8%, 23 episodes *E. coli* – 4.2% 1 episode	30-day mortality - 8.3% 90-day mortality - 20.8% Clinical cure at 30 days - 62.5% of episodes Adverse events - 16.7% patients
3	Caston et al., 2022	Bloodstream infection – 38.1% UTI – 35.4% IAI – 14.3% Pneumonia – 12.2%	CAZ-AVI (n = 189) BAT (n = 150)	After the diagnosis of infection (2 days, median)	*K. pneumonia* – 89.9% *Enterobacter spp.* – 4.8% *E. coli *– 3.2% Others – 2.1%	Mortality in patients with INCREMENT-CPE score of >7 points – 21.9% CAZ-AVI and 46.9% with BAT

**Figure 1:**
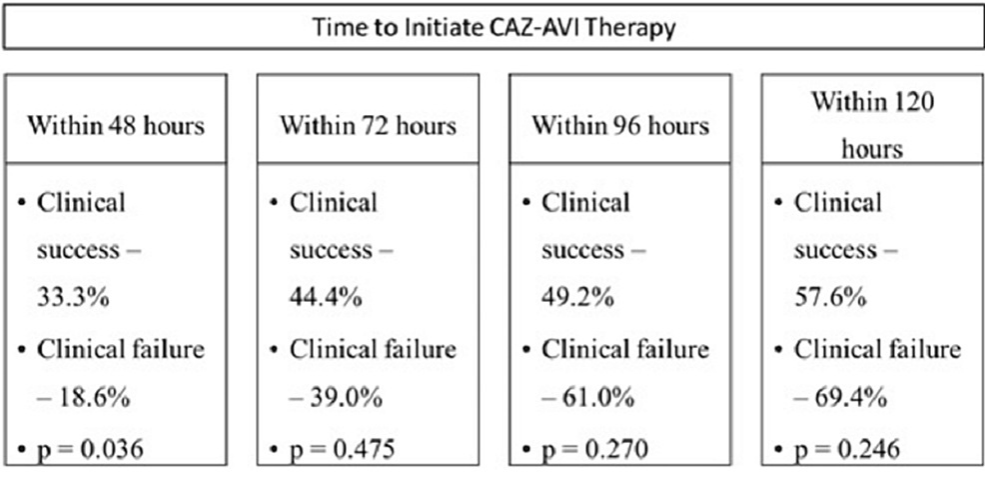
Clinical outcomes based on time to initiate ceftazidime-avibactam (CAZ-AVI) treatment post culture collection Jorgensen et al., 2019 [11]

Early use of ceftazidime-avibactam (within 48 hours of infection onset) has been associated with improved clinical outcomes [11]. The real-world experience with ceftazidime-avibactam, reported by Jorgensen et al., 2019 also highlights the advantages of using ceftazidime-avibactam at an early stage. The study reported data from 203 patients with MDR Gram-negative infections. the types of infections included respiratory tract infections (37.4%), UTI (19.7%), IAI (18.7%), and others (24.2%). Overall, 117 patients had CRE infections (63.2% with *K. pneumoniae* and 14.5% with *E. coli*) and 63 patients were infected with *Pseudomonas spp.* Among the 203 patients, 91 patients received ceftazidime-avibactam within 48 hours of infection onset. The composite clinical failure was reported to be 29.1% and 30-day mortality was 17.2%. In a group of 87 patients, ceftazidime-avibactam treatment was started within 72 hours. The clinical success rate when the treatment was initiated within 48 hours was 33.3% and failure was 18.6%. Similarly, the clinical success rate when the treatment was initiated within 72 hours was 44.4%, but the failure rate was 39.0%. For patients for whom the treatment was initiated within 96 hours and within 120 hours, the clinical failure rates were 49.2% and 61.0%, respectively, while the clinical success rates were 57.6% and 69.4%, respectively. Multivariate logistic analysis regression model for clinical failure showed that ceftazidime-avibactam given within 48 hours of culture collection was protective (adjusted OR: 0.409, 95% CI: 0.180 - 0.930) (Figure 1) [11].

Guideline recommendation of usage of ceftazidime-avibactam

Infectious Diseases Society of America (IDSA) has recommended ceftazidime-avibactam as a first-line treatment against OXA-48-like and KPC-producing carbapenem-resistant *Enterobacterales* for pyelonephritis or cUTI and infections outside of the urinary tract, in cases with proven *in vitro* susceptibility to ceftazidime-avibactam [60]. The Indian Council of Medical Research (ICMR) has recommended that ceftazidime-avibactam be used as a first-line treatment option against OXA-48-like carbapenem-resistant *Enterobacterales* [61].

Role of ceftazidime-avibactam in the Indian setting

In India, NDM and coproduction of NDM with OXA-48-like enzymes are the most prevalent mechanisms of CRE infections [62]. Ceftazidime-avibactam has been established to be effective in patients with comorbid conditions. The real-world evidence and clinical experience published consisted of patients with co-morbidities such as obesity, impaired renal function, diabetes, heart failure, liver diseases, malignancies [11], asthma, chronic pancreatitis [33], neurological diseases, bronchiectasis, etc. [31] among others. Sub-group analyses of REPROVE (ceftazidime-avibactam versus meropenem in nosocomial pneumonia, including ventilator-associated pneumonia) and RECLAIM (Efficacy and Safety of Ceftazidime-Avibactam Plus Metronidazole Versus Meropenem in the Treatment of Complicated Intra-abdominal Infection) studies were performed on data of Indian patients [26,28]. Both the analyses concluded that ceftazidime-avibactam was an effective alternative to meropenem in HAP and complicated IAIs (cIAIs) in Indian patients. The results of both the studies were in-line with the results of the overall results and the safety profile was consistent with the adverse effects previously reported for ceftazidime and cephalosporins [26,28]. The real-world studies from India have reported high susceptibility of tested CRE isolates to ceftazidime-avibactam [29] and also concluded that ceftazidime-avibactam is a viable option to treat patients with CRE infections [30].

A rise in carbapenem-resistant Gram-negative organisms has been observed worldwide. Carbapenem-resistant *P. aeruginosa*, *Acinetobacter baumannii* and CREs remain the major cause of hospital-acquired infections. This will inevitably lead to complicated treatment scenarios and more serious infections in vulnerable patient populations [63]. The current treatment options against MDR Gram-negative bacteria include polymyxins, aminoglycosides, tigecycline, carbapenems, fosfomycin, and newer β-lactam-β-lactamase inhibitors [64].

Tigecycline and colistin face challenges such as low plasma concentration and nephrotoxicity, respectively [5,65,66]. Colistin may be limited in its use due to its narrow therapeutic index, challenges with dose optimization, poor lung penetration, nephrotoxicity, and emerging antimicrobial resistance [61,65,67]. Antimicrobial resistance against colistin is emerging due to its rampant use. The susceptible category was removed from colistin by the CLSI indicating that some causative organisms might not respond to it owing to the unknown resistance mechanism [61]. Tigecycline has an expanded broad-spectrum activity which overcomes the resistance issues of tetracycline [68]. However, tigecycline has been reported to fail in achieving the required time curve and minimum inhibitory concentration ratio leading to treatment failure [69]. Tigecycline additionally faces challenges with regard to susceptibility testing due to inconsistency in results obtained from various antimicrobial susceptibility tests [70].

Ceftazidime-avibactam has been proven to be efficacious and, in some studies, non-inferior to conventional options in treating complicated infections [13,25,27]. There is limited literature available highlighting the importance of early use of antibiotics for treating infections caused by multidrug-resistant bacteria. However, clinical studies have stressed the association between delayed appropriate therapy and the risk of prolonged symptoms and treatment duration [53]. Prolonged hospital stays and treatment duration results in an increased economic burden for the patients and the healthcare infrastructures. It is hence crucial to alter the treatment practices from escalation strategies and adopt early and appropriate antibiotic therapy in patients with serious infections caused by Gram-negative bacteria [53].

## Conclusions

The increasing prevalence of antimicrobial resistance and infections caused by MDR Gram-negative bacteria jeopardize the current management strategies. Treating infections caused by CREs is more challenging owing to limited antimicrobial options. Ceftazidime-avibactam, a combination of the third-generation cephalosporin and a non-β-lactam-β-lactamase inhibitor, has been proven to be clinically efficacious in pivotal phase III non-inferiority trials as well as in real world settings. A decreased mortality rate was observed with early and appropriate use of ceftazidime-avibactam for managing infections caused by pathogens which are sensitive to ceftazidime-avibactam.

Furthermore, the use of rapid diagnostic tools can support prompt administration of effective therapy and help in reducing the morbidity and mortality associated with MDR infections. Ceftazidime-avibactam fits the role of an effective antibiotic with a favorable safety and pharmacokinetic profile. The early and appropriate use of ceftazidime-avibactam yields improved clinical outcomes for the patients whose profiles are suitable to receive early treatment. Further real-world evidence studies focusing on time of ceftazidime-avibactam treatment initiation are needed to ascertain the advantages of its early and appropriate use on a larger scale.
